# Assessing the Conceptualizations of Coping and Resilience in LGBTQ2S+ People with Cancer: Working towards Greater Awareness in Cancer Care

**DOI:** 10.3390/cancers16172996

**Published:** 2024-08-28

**Authors:** Sarthak Singh, Athina Spiropoulos, Julie Deleemans, Linda E. Carlson

**Affiliations:** 1 Michael G. DeGroote School of Medicine, McMaster University, Hamilton, ON L8S 4L8, Canada; sarthak.singh@medportal.ca; 2 Division of Psychosocial Oncology, Department of Oncology, Cumming School of Medicine, University of Calgary, Calgary, AB T2N 4N1, Canada; athina.spiropoulo1@ucalgary.ca (A.S.); julie.deleemans@ucalgary.ca (J.D.)

**Keywords:** LGBTQ2S+ health, cancer survivorship, strengths-based approach, interpretive phenomenological analysis

## Abstract

**Simple Summary:**

The cancer journey is often characterized by significant physical and mental health challenges for people with cancer during and after treatment; LGBTQ2S+-identifying people with cancer may face additional issues surrounding their identity and discrimination in health care settings. However, there is limited research that explores the unique experiences of LGBTQ2S+ people with cancer. This study investigates the resilience of LGBTQ2S+-identifying people with cancer to understand how they experienced and coped with cancer. Most participants’ cancer journeys were characterized by a ‘Second Coming-Out’ phenomenon, where LGBTQ2S+ people with cancer use coping strategies, similar to those used when coming out, to produce resilience throughout their cancer journey. Identifying this phenomenon is critical to providing comprehensive care for LGBTQ2S+ people with cancer that draws on their unique strengths. We propose the LGBTQ2S+ Cancer Care Model where the individual is centered and the influences of personal support, professional support, and queer representation are integrated in an accessible and clinically relevant way.

**Abstract:**

People with cancer may suffer negative psychosocial outcomes due to the challenges of cancer. LGBTQ2S+ people routinely experience negative psychosocial outcomes in health care settings, but have showcased resilience in the face of discrimination; however, this has never been studied in a cancer context. Thus, this study aims to assess coping and resilience in LGBTQ2S+-identifying people diagnosed with cancer using a strengths-based approach. A qualitative exploratory design was used. Ten self-identified LGBTQ2S+ people who have completed their cancer treatment were recruited. Participants completed clinical, health, and demographic questionnaires and, subsequently, semi-structured qualitative interviews. Conceptualizations of coping and resilience in the semi-structured interviews were analyzed using interpretative phenomenological analysis (IPA). Participants were members of various gender identities and sexual orientations. In addition to identifying needed LGBTQ2S+-specific resources, four narratives emerged: support networks, regaining control in life, conflicting identities, and traditional coping methods. Most participants’ cancer journeys were characterized by a ‘Second Coming-Out’ phenomenon, where LGBTQ2S+ people with cancer use coping strategies, similar to those used when coming out, to produce resilience throughout their cancer journey. This work provides exploratory insight into LGBTQ2S+ people with cancer, but more research is required with a larger sample.

## 1. Introduction

The psychological and social outcomes faced by people with cancer can result from the unique and dynamic challenges of the disease itself, treatments, or the survivorship experience [1,2]. A meta-analysis demonstrated that anxiety was significantly more prevalent in survivors of cancer than in healthy controls, [3] and more than one-third of Canadian survivors of cancer experienced symptoms of distress, including fatigue, depression, and anxiety [4].

### 1.1. Current Considerations and Experiences of LGBTQ2S+ Cancer Patients

There is a heightened risk of certain cancer types in LGBTQ2S+ populations, partly due to the medical mistrust these communities face when dealing with health practitioners; in a study using a large Californian dataset (*n* > 100,000), it was found that gay and bisexual men were almost twice as likely as heterosexual men to have a cancer diagnosis [5]. For cancers that can be caught earlier or prevented by routine screening or checkups with a health care worker, risks can be exacerbated. In Belgium, a mixed-methods study of 50 transgender women showed that there was a fear of going for a mammogram within this population, which increased the risk of breast cancer [6]. Despite these elevated risk factors, there continues to be limited psychosocial oncology research which centers on LGBTQ2S+ people with cancer, and even in broader studies on the general cancer population, sexual orientation and gender identity are rarely considered in the analysis [7].

Emerging research on LGBTQ2S+ survivors of cancer reveals startlingly poor psychosocial outcomes. In a cross-sectional study of mental health outcomes in gynecologic survivors of cancer, lesbian and bisexual participants experienced higher rates of anxiety, depression, and post-traumatic stress disorder, in contrast to heterosexual participants [8]. These populations also face poorer access to care; in a study of over 70,000 cancer patients, of which 2000 were sexual and gender minorities, compared to heterosexual women, women who were sexual minorities had significantly more access deficits (according to the BRFSS survey) which was associated with poorer physical and mental quality of life [9]. Moreover, LGBT2S+ cancer patients report that, when trying to involve their partners in their recovery journey, interactions with nurses were stressful and anxiety-inducing, and ultimately led to increased feelings of isolation [10]. Specifically, for LGBTQ2S+ survivors of cancer, LGBTQ2S+-specific support groups were often inaccessible and general support groups created heterocentric and unwelcoming environments that only furthered isolation and depressive feelings [11]. Overall, LGBTQ2S+ people with cancer can experience harms beyond the scope of, and often unique from, heterosexual cis-gendered cancer survivors.

### 1.2. Strengths-Based Research: Coping and Resilience

A significant limitation of the current state of LGBTQ2S+ psychosocial oncology research is that it has been predominantly deficit focused. Exclusively highlighting the risks or negative factors that ultimately lead to poorer health is problematic as that framing of LGBTQ2S+ health research influences the access and use of health resources by LGBTQ2S+ service users [12]. Moreover, deficit-focused research can be demoralizing to LGBTQ2S+ individuals and further lead to poor health outcomes [12]; instead, research that aims to be strength-focused, with a particular emphasis on resilience [12], can be useful and motivating to the community. Strength-focused research underscores the need to recognize how communities or individuals face and overcome their challenges, particularly through self-led means [12], and has been applied successfully to LGBTQ2S+ research [13]. Within the general cancer population, it is understood that social protective factors that build resilience can have a positive impact on quality of life and decrease negative psychosocial outcomes [14], so exploring these strengths-based outcomes in LGBTQ2S+ people with cancer may be beneficial.

Coping is also a central facet of strengths-based work. Coping, from a clinical perspective, considers adjustment or adaptation, notably how one responds to ongoing demands and concerns [15]. Coping can be both adaptive, something that brings about relief and decreases stress, or maladaptive, something that may bring about additional harm [15]. One of the only studies to mention coping in LGBTQ2S+ survivors of cancer found that gay participants worried less about infertility being a risk and were, incidentally, better able to cope with problems experienced in their relationships due to the burden of cancer [16]. No research has directly asked LGBTQ2S+ people with cancer to conceptualize coping and resilience in their cancer journeys, and research has yet to consider how LGBTQ2S+ people with cancer attain resilience in survivorship at all.

Resilience is also a central component of strengths-focused research, encapsulating how people recover or bounce back from adversity [17], within the context of their personal and environmental protective or risk factors [18]. Serving not only as an endpoint, resilience in cancer research is also seen as an iterative process, whereby survivors of cancer recalibrate their responses to, and understandings of, adversity to become resilient [19]. Throughout their cancer treatment or journey, people may demonstrate resilience in the form of positive affect or decreased fear of reoccurrence; however, resilience is still deeply individualized and often related to initial levels of distress in relation to their cancer diagnosis [19].

### 1.3. Primary Study Aims

The present study utilizes a critical inquiry approach, grounded in an intersectional framework, to explore what the cancer survivorship journey looks like for LGBTQ2S+ people with cancer and how they cope or attain resilience through that journey. The present study has three objectives: (1) examine the ways in which LGBTQ2S+ survivors of cancer conceptualize their cancer experience; (2) explore factors that support coping and resilience throughout cancer treatment and survivorship for LGBTQ2S+ people with cancer; and (3) explore coping and support needs that LGBTQ2S+ people with cancer would have liked to see during their recovery process.

## 2. Materials and Methods

### 2.1. Study Design

The present study uses a qualitative exploratory design. Following eligibility screening and the provision of informed consent, participants completed online questionnaires and one virtual semi-structured interview, which ranged from 30 to 90 min. Exploratory quantitative findings were used to inform the development, facilitation, and analysis of interviews. The present study was approved by the Health Research Ethics Board of Alberta (HREBA)–Cancer Committee (CC): HREBA.CC-22-0256.

#### Utilizing a Patient Oriented Approach

LGBTQ2S+ perspectives from lab members and patient partners were incorporated throughout the research process—from developing research questions and methods to data collection and the reporting of results—which has been seen to increase patient participation and improve outcomes and the applicability of research findings in patient groups [20]. LGBTQ2S+ groups are underrepresented in health research and incorporating patient perspectives allowed for an informed and respectful approach to the research project.

### 2.2. Participants

Ten participants were recruited from across Canada using convenience and targeted sampling. Social media was one medium used for recruitment, sending out information to cancer-related social media groups such as YACC (Young Adults Cancer Canada), Wellspring, and Queering Cancer, and LGBTQ2S+ health groups, such as Rainbow Health Ontario. Promoted posts were used on Twitter and in other online cancer groups and forums. Participants were also recruited through promotional pamphlets and information slides at the Tom Baker Cancer Centre and Holy Cross Centre in Calgary. Recruitment also took place through the Queering Cancer email network. Queering Cancer operates nationwide, and due to the virtual nature of the study, patients from across the country were able to participate and were targeted for recruitment. Potential participants reached out to the student researcher directly to arrange a time for screening and to discuss consent.

The inclusion criteria for participants were as follows: (1) diagnosed with any type of cancer (infiltrating malignant tumor) and who had completed cancer treatment within the past five years; (2) no instances of cancer recurrence; (3) self-identified as member of the LGBTQ2S+ community; (4) have access to a technological device and internet connection; and (5) be able to read, write, and speak in English. Notably, participants who were questioning their identity (i.e., unsure or still in the process of identifying their sexual orientation or gender) were also eligible, as most current definitions of the LGBTQ2S+ community incorporate the ‘Questioning’ identity.

#### Sample Size Justification

The sample size (*n* = 10) was determined based on the adequate sample size for interpretative phenomenological analysis, which IPA scholars typically postulate should be between 4 and 10 participants to allow for a sufficient but not overwhelming amount of data [21]. A sample size of 10 was chosen for a more rigorous analysis, and it was seen that even at around 7 interviews, data saturation had been achieved.

### 2.3. Measures

Study demographics and clinical data: Clinical (e.g., cancer diagnosis, date of diagnosis, treatments received, etc.) and demographic (e.g., income, occupation, ethnicity, etc.) data were collected (Appendix A). In alignment with previous LGBTQ2S+ research, participants were asked to identify their gender, with nine options presented (Man, Woman, Non-binary, Transgender, Genderqueer, Genderfluid, Agender, Two-Spirit, and Other (please specify)), as well as their sexuality, with 10 options presented (Heterosexual, Lesbian, Gay, Bisexual, Queer, Asexual, Pansexual, Demisexual, Questioning, and Other (please specify)). None of these questions required responses.

A semi-structured interview guide was collaboratively developed by integrating quantitative results and the existing literature (Appendix B). All interviews were conducted by the first author (SS), recorded with consent, and later transcribed.

### 2.4. Data Analysis

Information obtained via semi-structured interviews was audio recorded and transcribed verbatim. The content was coded in NVivo using interpretative phenomenological analysis (IPA) to explore each of the objectives. IPA involves the primary researcher first reviewing the data and understanding the phenomenon (‘resilience and coping in cancer survivorship’) while bracketing out personal beliefs through the practice of reflexivity [21]. The first author (SS) coded the transcripts for units of meaning which were of importance to the phenomenon. A second coder (AS) independently analyzed transcripts in the same process. The two coders then met to consolidate the codes into fewer codes and eventually the key themes and subthemes. A theme summary was sent back to a subset of participants for validation.

The credibility of the semi-structured interviews was addressed using frequent peer review (consensus approach between two coders) to triangulate the data and participant review authenticity checks. Additionally, coders (SS and AS) acknowledged their positionality through reflexivity statements included in Appendix C.

## 3. Results

### 3.1. Sample Demographics

Ten people who had survived cancer participated in the present study. Participants were, on average, 36.30 (±13.54) years of age. In terms of sexual orientation, participants identified as bisexual (40%), lesbian (30%), gay (10%), and queer (20%). In terms of gender identity, participants identified as men (20%), women (40%), non-binary (20%), transgender (20%), and 2-spirited (10%). It is important to note that one participant identified as both transgender and a man, hence why the percentages do not equate to 100%. As seen in Table 1, participants were ethnically First Nations (10%), Black (30%), South Asian (10%), South-East Asian (10%), and White (30%). Most participants were university educated (70%), living with a partner (60%), and living in urban centers (80%). 

### 3.2. Qualitative Findings

Objectives 1, 2, and 3 were addressed by the ‘Second Coming-Out’ phenomenon, wherein participants drew on coping strategies they developed when coming out as queer to navigate cancer treatment and survivorship. Four primary narratives comprise the ‘Second Coming-Out’ phenomenon: (1) having a support network, (2) regaining control, (3) conflicting identities, and (4) traditional coping methods. Objective 4 was addressed in a separate theme, in which future considerations for cancer care that are sensitive to the unique needs and lived experiences of LGBTQ2S+ patients are explored.

#### 3.2.1. Experiencing Support Professionally and Interpersonally

For participants, having well-integrated and meaningful personal and professional networks made them feel less alone and more supported. Conversely, a lack of such a support network left participants feeling misunderstood and anxious about if they were receiving adequate care.

*Professional Support Network*. Having positive experiences with health care providers was meaningful and supported them through the otherwise tumultuous aspects of their care journey, particularly if they were explicitly queer-affirming or trauma-informed in their care.

“It was also very trauma-based, trauma-informed. As somebody who has trauma, most queer people do, but as somebody who has trauma and also had trauma around this biopsy, [the health care provider] even sent me to places where they would not misgender me.”

(S10)

Two participants recalled having outright judgmental health care providers who appeared uncomfortable providing care, due to the participant’s gender identity, which had quite negative implications for the participant.

“I think most of the doctors lack understanding and sensitivity, they don’t know how to deal with [LGBTQ2S+ people with cancer], they don’t know how they can handle us.”

(S4)

Having care professionals leave questions unanswered or appear unapproachable made the participants uneasy and feel that their care was being dismissed. However, they also noted this may have been a consequence more of health care providers’ busy schedules rather than any anti-queer bias, but one participant noted her health care provider would avoid questions asked by her partner, who identified as a woman, routinely. Participants noted that upon finding new more supportive health care providers they felt their care improved.

*Personal Support Network*. More than half (*n* = 6) of the participants mentioned having their biological family, particularly their parents, involved in the treatment and survivorship journey, in cases where their family could be a force that helped them navigate health care settings and became a pillar of support for them. Conversely, family could also be difficult to manage because of their overbearing nature and even underlying homophobic subtext. For those whose biological families were not a calming presence or even part of their journey at all, there was reliance on “chosen family” and friends. This ability to cultivate a “chosen family” for support was a skill participants had developed when coming out.

Partners, for those who had them, were also a major source of support and often reminded the patients of concerns or questions they meant to ask. Having a visibly queer partner did bring some anxiety as to how health care providers would react, but ultimately this was not a major issue. Friendships were most supportive when friends could anticipate and adapt to the needs of the participant.

“I talked to my friends. I talked, I talked, and I talked. And I was uninhibited about what I said. I just let it all out. Let it all out, release it. I wasn’t looking for advice, and they knew that. I wasn’t looking for how could they give me advice? What could they say? But they were listening. They were good sounding boards, and that’s what I needed for me, good sounding boards.”

(S6)

#### 3.2.2. Regaining Control in Life

Participants discussed how cancer and the rigorous treatment regimen took away autonomy and they aimed to find control in other aspects of their life to cope. While the specific strategies participants employed were often tailored to the cancer context, participants’ coping tendencies were informed by their approach to coping when coming out as LGBTQ2S+.

*Functionality*. The most important way for participants to feel a sense of autonomy and control in their life was to remain highly functional even through the most physically and mentally taxing parts of their cancer journey.

“I think coping is getting along with a certain condition, with a certain thing.”

(S4)

One of the most common ways of remaining functional was actively participating in their treatments, including going to appointments, proactively asking questions, and inquiring about options for survivorship care. By contributing to their care plan, participants felt more productive, but also that they received more optimal care.

Three participants indicated that focusing too intensely on functionality limited their ability to feel secure in their other identities and explore their lives beyond cancer. Among these participants, often there was a dissociation from reality and lack of cognizance of what was actually occurring to them or what activities they were undertaking, such as school or work, even if they were quite successful in them.

“Even like I said, with my discussion of my identity, it kind of feels like all those years [during cancer treatment?] were sort of just spent lived in like a weird dream, rather than something that I went through.”

(S1)

*Physical Wellness*. One part of regaining control was working towards controlling their body through physical means; this included engaging in intentional physical activity in the form of weightlifting, walking, or running. A recurring theme was the benefits of physical activity (outside or in nature specifically) and nutrition.

“Maybe eating a healthy diet. I think, by that I was taking care of myself, and maybe finding ways to manage my stress because sometimes I was stressed, and I think it was not good for my health.”

(S4)

In some cases, the notion of controlling one’s own body transitioned into disordered thinking and body image issues as participants attempted to compensate for the lack of control over cancer and outcomes.

“So, I struggled a lot with really restrictive eating. I struggled with just really severe control over my body and body image. Just control and a lot of avenues in my life became a huge way of coping with illness. But I think in my mind, I saw it as just something else. I was like, no, you just—you know, you probably don’t have an eating problem or image problems and I really didn’t make the connection.”

(S1)

#### 3.2.3. Making Meaning of Conflicting Identities

Being a cancer survivor was an added identity for participants that compounded on but, at times, also conflicted with their queer identity. Particularly with sex or gender-based cancers (i.e., cancer types that were typically associated with a specific sex such as breast, ovarian, or prostate cancer), the cancer felt at odds with their queer identity, and they felt excluded from traditional survivorship spaces. Representation was important in creating an added level of comfort and coping through the survivorship process, but queer representation was significantly lacking within cancer or health care spaces.

*Identity Through the Cancer Process*. Going through cancer often meant putting identity exploration and discovery on hold for several participants (*n* = 6), wherein being a cancer patient and survivor was the main identity to prioritize. This meant the participants had less time and effort to dedicate to honoring their LGBQT2S+ identity. Sex-based cancers, such as uterine or breast cancers in females and prostate cancer in males, particularly had an impact on participants, as the highly gendered facets of these were at odds with participants’ queer identities. Being a queer survivor felt doubly isolating, as both a cancer survivor and a queer person.

“Cancer is a very alienating thing, because it’s hard to understand if you’ve not gone through it. And for a lot of people, and certainly for a large portion of my life, being queer was a very alienating thing. And adding the two together is, I was just going say it’s doubly hard.”

(S2)

For participants who were survivors of breast cancer, reconstruction was considered the norm by providers and within the literature and they felt isolated in choosing not to take up that option.

“Everything I’ve read at first about mastectomies was about like deciding about reconstruction. And I just, I wanted to hear from people who had made the choice not to have reconstruction, or people who—I wanted to hear from queer people, I wanted to know how someone who wasn’t concerned about femininity or someone who might be gender fluid felt about having a very strongly gendered cancer.”

(S2)

For transgender participants, sex-based cancers were particularly tough as providers sometimes did not adequately understand how to provide care and participants often felt like outsiders within treatment and survivorship spaces. Coming out to providers led to confusion or outright comments by others or providers about how their gender identity did not match the cancer they were experiencing, and this was incredibly isolating and negative for transgender participants.

“So, maybe—let me use myself—let me just explain it to you. So, I had breast cancer, and I was trying to access healthcare. So, I went to the first doctor, and he was like, “you know, you, you, you look like you are a man, and you have breasts”, so things like that.”

(S4)

Participants who did not have a sex-based cancer felt less distress and conflict with their LGBTQ2S+ identity as there was often no need to disclose gender identity or sexuality to health care providers; this allowed for a sense of comfort, though participants mentioned going “back into the closet” did bring up somewhat traumatic memories or guilt about coming out when they were younger.

“All the health professionals that I encounter, not that every one of them knows that I’m gay, I mean, some of them don’t need that information, unless I disclose that to them.”

(S3)

For one participant who had a non-sex-based cancer, he felt that going through his cancer made him more comfortable in his gender identity; this was because he had mostly positive reactions to coming out in the hospital setting. He was the only participant who noted this. 

*A Desire for Representation*. Participants noted that while LGBTQ2S+ representation was lacking throughout hospital resources and information, it was particularly lacking in the cancer space. With any of the pamphlets or information guides, there were no references to LGBTQ2S+-specific issues. Beyond hospital resources, however, even the general literature about their cancer was also highly gendered or exclusive of their gender identity. For example, for participants who survived breast cancer, who identified as queer, most of the breast cancer literature was heavily focused on femininity and this aspect of female beauty, which participants did not feel fit their identity.

“Well, it was kind of frustrating because I knew there had to be some books out there that weren’t about becoming beautiful again, after breast cancer or whatever. There’s a whole hell of a lot of pink books, they’ve all got cupcakes on the cover, because cupcakes apparently are the pastry that looks most like breasts, you can put them on the cover of a book. So, I was doing a lot of eye rolling when I was trying to look.”

(S2)

The “pinkwashing”, as it was referred to, made participants feel even further excluded or simply made them feel as though their survivorship path was less worthy. On the rare occasion that queer literature was available, which was only found after extensive searching digitally by the participant themselves, it was incredibly helpful and particularly made participants feel less alone. The sense of inclusion and belonging felt by having such literature was important.

“It’s very much parallel to the feeling you get when you recognize queer experience right? And so, reading the books I would have that same experience, but a sort of a double I see you, in the sense of, oh, I see you as a queer person. I see you as a queer person with a highly gendered cancer that is marketed in ways that are, super tied to femininity, which is a thing that many queer women who have breast cancer have had to struggle with already at some point. And so, it was just that it’s—it was exactly like being out in the world and encountering someone where there’s that moment of acknowledgement.”

(S2)

Beyond LGBTQ2S+ representation generally, participants from a racialized background noted that even more specific representation was needed and that aiming to find their identity within whiter homogenized LGBTQ2S+ resources was tough. The unique intersection of their cultural identity with being LGBTQ2S+ and a cancer survivor put them in a position that made them feel very alone and any sort of story about others like them would have made them feel more connected and, therein, more secure within their cancer journey.

“Even in kind of, for example, queer-related resources for oncology. Maybe having some nuance for minority groups within that. So, I obviously am Indian, like a person of color. So, I think having something with this. I know, now we’re looking for a subgroup within that gay survivor subgroup, and that Indian gay survivors are going to be very few people, I’m sure, but just to have a resource or something.”

(S9)

#### 3.2.4. Drawing on Existing Coping Strategies

Participants relied on many adaptive and maladaptive traditional coping strategies that they initially accessed when coming out as LGBTQ2S+. 

*Avoidance Coping*. Avoidance coping primarily centered on ignoring the reality of cancer; in some cases, this was paired with participants trying to simply focus on other parts of their life, but sometimes it was simply a cognitive distortion wherein the harms of cancer were not mentally registering to the participant. This resulted in the detrimental psychosocial effects of the cancer, manifesting later on with increased intensity.

“I guess in my awareness of my cancer journey, I would say there’s definitely been a marked difference in the point in time when I recognized that cancer was something that actually was a problem.”

(S1)

*Acceptance*. Another form of coping used by participants was that of accepting the reality of their cancer. This meant sometimes understanding that their life was not going to return to what it once was and that they would have to adapt to a ‘new normal’.

“Yeah, just the sort of sense of, I will have to shift my sense of self in order to sort of accept what my limits are on a given day. So that was a big part of it for me, during treatment.”

(S2)

*Self-Advocacy*. For many participants, self-advocacy was empowering, particularly by allowing access to resources they required for mental and physical health support. Participants also self-advocated by finding resources, often through the internet, and joining support organizations that linked them with further resources.

“I joined the Thyroid Cancer Association as soon as I found out, so they had lot of literature, researchers that were extremely helpful. Pamphlets, free information, so I did my own research a lot.”

(S3)

However, for some participants, having to be their own self-advocate felt as though they were unsupported in the system and that was emotionally taxing. In particular, when a participant became a spokesperson within cancer circles about their journey, this was challenging because it felt as though others relied on her efforts and that she was a role model to others. Even though she was rehashing her own traumas and did not always feel strong enough to share her story, others viewed her as superhuman and very strong, and she did not want to let them down, which consequently was debilitating to her mental health and coping.

“I think it kind of became this weird cycle of—I would share my story. And obviously, I would kind of come across in this way where it’s, “I’m getting through it, and I’m fine”. I’ll get a lot of positive response and praise on that, where it’s like, “oh, you’re so strong and, I can’t believe you’re able to get through it like this”. And it kind of became this, feedback cycle where it’s, the more I repress my emotions, the more positive response.”

(S2)

*Mental Wellness Resources*. Working towards mental wellness was very important to be able to cope with the cancer. This fit in with conceptualizations of resilience that participants had, notably that resilience was this self-desire to flourish in the new adjusted circumstances of surviving cancer and that there was an important aspect of feeling healed that exemplified resilience.

“I think there’s definitely kind of an awareness and a healing component to resilience to where there actually is a part of you that does deal with the problems you’re facing.”

(S1)

The most critical mental wellness coping strategy was that of therapy or counseling resources. All participants (*n* = 10) saw a therapist, and everyone noted the positive impact it had on their well-being and survivorship journey. Typically, effective therapists were queer-affirming, trauma-informed, and specialized in psychosocial cancer care.

“I started seeing a therapist back when I was at the Children’s too. So, their psychosocial oncology team, I had someone with me, I think got assigned pretty soon after my cancer journey started there. I mean, yeah, I think having a person there, that’s not part of the rest of your life is just extremely helpful and kind of navigating school’s challenges, especially someone who’s knowledgeable in cancer.”

(S1)

#### 3.2.5. Current Gaps in Service Provision

To assess objective 4, participants detailed what supports could have been available to them that would have increased support and coping during their cancer care journey. These suggestions included formal and informal changes to both health care and social contexts that participants felt would make survivorship a resilient and positive journey for them. Three subthemes summarized these considerations: a centralized system, LGBTQ2S+-specific resources, and the cultivation of authentic relationships.

*Centralized System*. In response to concerns about the lack of navigability and centralization of care within the system, participants noted the need for a patient navigator that connects people with cancer to various traditional and complementary mental and physical health resources needed from the beginning of their cancer diagnosis. Having connections to cancer-specific therapists, such as a psychosocial oncology psychologist, was important.

“And I was saying to my therapist, I said there needs to be a rehab. After you are all done, and they send you off, go fend for yourself, there needs to be a rehab. You need to get a dietitian in there. You need to get a physical therapist in there. You got to get a massager in there. You need a mental health person in there. There needs to be a rehab center for after cancer.”

(S6)

*LGBTQ2S+-Specific Resources*. An important need participants identified was for health care professionals to have a greater understanding of LGBTQ2S+ people and their specific concerns. Several (*n* = 5) participants noted that they had at least one health care professional who they felt did not have accurate or adequate understandings of queer people. Participants noted that having health care professionals better understand LGBTQ2S+ people and LGBQT2S+ cancer care would make survivors’ personal journeys through the cancer system more bearable.

“One thing that I would like to see maybe is understanding of healthcare providers and the support team at the hospitals. Maybe—if they can gain more understanding of, of LGBTQ individuals, I think it can be better. That’s the only thing I think maybe they’re lacking.”

(S4)

Another key need participants mentioned was increased representation of LGBTQ2S+ people in the hospital and cancer literature. While more superficial pictorial representation is nice to see, it was noted as most impactful if there were direct digital links or written-out pamphlets redirecting survivors to LGBTQ2S+ cancer resources, such as Queering Cancer. As well as this, having queer stories within cancer literature was a strong desire for multiple participants, as they felt it would help them feel less alone in the otherwise isolating journey of being a queer survivor.

“So later on, I went back and I told that nurse about Queering Cancer. And she said, well, thanks for letting me know, that’s really great. I’ll take a look at it, we have to take a look at all of our resources and sort of, vet them first to decide what we include in our official patient information... But I was really disappointed because my last appointment in October they had updated the brochure, and they did not have that included. And that was a shame, I thought, because even though the proportion of their patients who are queer is probably relatively small, we’re still there. And we still need information and stories and that kind of thing. So, I wish that had taken me up on that.”

(S2)

As well as this, participants noted that more research needs to be carried out in the field of LGBTQ2S+ cancer care, particularly noting that having researchers or others take interest in the negative and positive aspects of their cancer journey felt fulfilling. 

*Cultivating Authentic Relationships*. Lastly, participants mentioned the need for authentic relationships both in professional and personal domains to support their coping. Participants noted that having personal support networks that allowed them to feel the full range of grief and emotions that cancer brought would have let them approach their cancer journey in a more psychosocially positive way.

“I think definitely someone telling me you can grieve about this, you’re allowed to, you know, want these resources and you’re allowed to be upset and you’re allowed to be affected. I think for me, that’s probably the only thing that might have, kind of gone through to me to say, okay, maybe I will look at some of [these resources].”

(S1)

## 4. Discussion

Participants in the present study experienced a ‘Second Coming-Out’ phenomenon in terms of how they fostered resilience. Participants also identified that accessing LGBTQ2S+-specific resources and cultivating authentic relationships with health care professionals was paramount to successfully navigating cancer treatment and survivorship.

### 4.1. Conceptualization of Coping and Resilience

Coping was defined by participants as heavily relating to functionality, that is, being able to continue with ongoing activities and function at satisfactory levels despite the ongoing challenges of cancer. This connects to the construct of coping in the literature, which is often associated with adjustment or adaptation [15].

Resilience was heavily tied to notions of mental wellness and cultural context. Participants understood resilience to be a mental construct which incorporates elements of healing, recovery, and self-betterment. Recalibration, that is, bouncing back from adversity, is theorized to be a fundamental component of resilience [19]; however, this was not explicitly discussed by participants in the present study. Despite this, the idea of recovery and healing both connect to the re-examination of cancer and recalibration of attitudes towards cancer, meaning there is some overlap with established theoretical definitions of resilience [19].

### 4.2. Exploring the ‘Second Coming-Out’ Phenomenon

**Figure 1:**
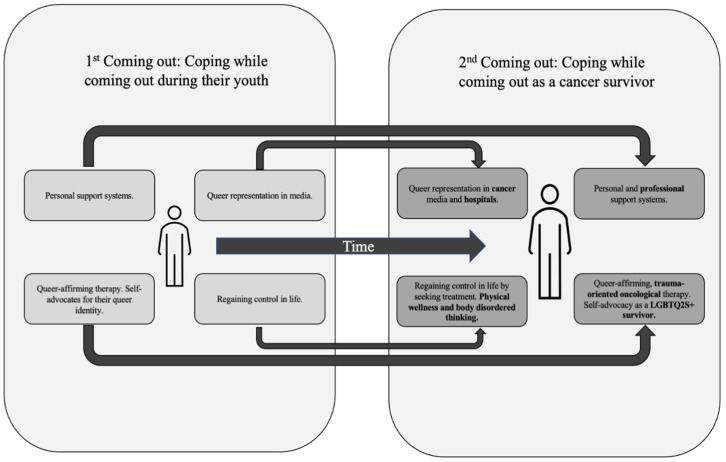
Second Coming-Out’ Model.

Participants’ experiences reflected a ‘Second Coming-Out’ phenomenon, wherein LGBTQ2S+ people with cancer experienced stressors similar to when they came out during their teenage or adolescent years in a new cancer context, as depicted in Figure 1; moreover, both the adaptive and maladaptive coping strategies used when coming out were drawn on once more during cancer treatment and into survivorship.

#### 4.2.1. Support Systems

Personal support networks could serve as positive or negative coping mechanisms during LGBTQ2S+ people with cancer’s initial coming out process. Having family or friends who were willing to adapt to the needs of the LGBTQ2S+ person with cancer and fill needed roles in the participant’s life was important, just as it was during their coming out. This aligns with the current literature as social support networks have demonstrated protective effects against discrimination in LGBTQ2S+ youth [22,23]. Moreover, research for a general breast cancer cohort showed that as social support declined, emotional well-being also declined [24].

Having centralized and well-connected care teams made the cancer treatment and survivorship journey more comfortable for participants in the present study. This is informative, as past literature on LGBTQ2S+ oncology has shown that queer survivors of cancer may experience a fear of health care professionals and potentially discriminatory practices [25]. Even in general cancer survivor populations, a lack of communication between health care professionals and uncoordinated care has been noted as inefficient and reducing the standard of care received [26].

#### 4.2.2. Regaining Control

In the present study, remaining highly functional to regain control through distress was seen during coming out and this repeated during cancer survivorship. While this allowed for greater adherence to treatment, participants noted a decrease in emotional vulnerability, which negatively impacted coping. For participants, a new element of physical wellness was added in, which encompassed the positive coping elements of better nutrition and physical activity, and the negative elements of body image disordered thinking. Physical activity and its ability to lower negative physical and psychosocial outcomes for people with cancer has been well documented [27]. This has not been evaluated in LGBTQ2S+ cancer populations and is worth assessing considering that non-cancer LGBTQ2S+ populations routinely have higher body image disordered thinking [28]. How to tactfully promote physical wellness behaviors, in a way that is inclusive for LGBTQ2S+ people with cancer, to gain more control in their life without causing dysphoric thinking, is an important area to consider in further research.

Traditional coping strategies were used by participants when coming out initially and again when they came out in health care contexts and moved through their survivorship journey. In particular the concepts of denial, which had negative implications, and acceptance, which had positive implications, were seen as the participants in the present study came out of the closet and later as they dealt with their cancer diagnosis. Acceptance of both cancer and survivorship has shown positive psychosocial benefits for people diagnosed with cancer in general [29] and is considered an important part of coping with a cancer diagnosis, now applicable to LGBTQ2S+ survivors as well.

#### 4.2.3. Queer Representation in Media

Research has shown that LGBTQ2S+ representation in the media has positive impacts on LGBTQ2S+ people and increased awareness and acceptance in non-LGBTQ2S+ populations [30]. This was reflected in the present study, as participants noted that when they encountered queer representation, usually in social media or a book, it had an incredibly positive impact on them and helped them better understand their condition and achieve resilience. In the cancer context, the need for better representation is particularly important in sex-based cancers, such as breast, uterine, and prostate cancers, where LGBTQ2S+ identities may conflict with the care received. Sex-based cancers have been the focus of LGBTQ2S+ survivorship research; however, the role of media representation has never been explicitly addressed. It is also of note that, while not mentioned by participants here, cancers of the cervix, anus, and oral cavity may also be important, as they may relate to gender identity and/or sexual behaviors.

#### 4.2.4. Queer-Affirming Support and Self-Advocacy

In the present study, LGBTQ2S+ survivors of cancer were regularly required to be self-advocates, similar to how they felt as young queer people in the world. Self-advocacy has been seen in general cancer survivor groups and has positive ramifications for achieving better outcomes in care [31]. However, as the findings from this study indicate, constant self-advocacy can be harmful as it demands that individuals be extraordinary to receive ordinary care; the disability literature defines this within the supercrip model, that is, when patients feel they have to do more, often to prove they are still worthy despite their disability [32].

Accessing therapy was brought up as a positive coping mechanism and was used by all participants in this study, comparatively much higher than what is seen in the broader cancer population (17.6%) [4]. Therapy was used frequently by participants when they were coming out and was still important in the survivorship journey, but it was reinforced that such therapy was most beneficial when it was cancer-specific. Participants noted psychosocial oncology mental health practitioners were the most effective and this stemmed from their understanding of cancer and importantly the interactions of cancer with LGBTQ2S+ issues. Participants’ indications that more must be done by health care professionals to become LGBTQ2S+-informed and queer affirming in their care are supported within the literature. A study showed that less than half of cancer providers correctly answered LGBTQ2S+ knowledge questions [33]. Despite this, health care professionals did show positive interest in becoming more aware of how to better treat LGBTQ2S+ communities [33], indicating that perhaps having more cancer-specific LGBTQ2S+ education for health care professionals, and psychosocial oncology workers specifically, may be of particular benefit.

### 4.3. Implications and Future Directions: A Model for LGBTQ2S+ Resilience-Focused Care

**Figure 2:**
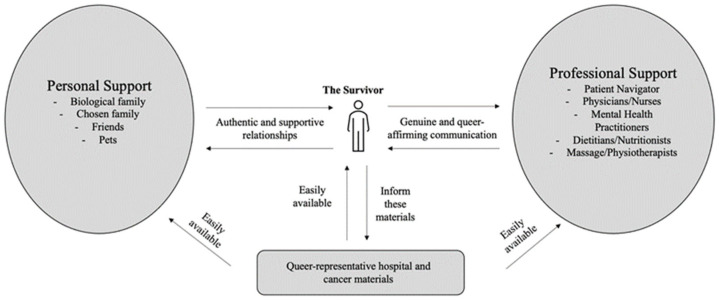
LGBTQ2S+ Cancer Care Model. Note: the LGBTQ2S+ person with cancer is bilaterally connected to their personal and professional support networks, and they inform queer-representative cancer materials which are available to all three stakeholders.

The present study contributes to understanding how LGBTQ2S+ people experience cancer survivorship and what coping mechanisms or pathways to resilience are most effective. The LGBTQ2S+ Cancer Care Model is a resilience-oriented care model that centers the patient (see Figure 2).

Findings from the present study demonstrate that LGBTQ2S+ people with cancer benefit from being surrounded by meaningful professional and interpersonal support networks that recognize the needs of the survivor of cancer and adapt to those needs. Having a multidisciplinary team of health care professionals that provide standard and complementary care decreased participants’ anxiety surrounding cancer, reduces the need for overbearing self-advocacy, and helps to promote positive coping behaviors in the domains of physical and mental wellness. These health care professionals should move towards being queer-affirming providers, with an understanding of LGBTQ2S+ issues and being trauma-informed in their care. Some of this gap should be bridged through the medical education system itself, with increased curricular and extracurricular components about queer health. Just around half of participants did not have providers who they felt had adequate understandings of the unique needs of queer health, and thus this is a vital gap to fill.

Based on the present study, we recommend that in hospital-provided resources, there should be more integration of LGBTQ2S+ resources or representation and this should be available to the entire network of people supporting LGBTQ2S+ people with cancer. These resources ideally would be easily available and accessible and advertised, so patients do not have to search extensively to find them. The LGBTQ2S+ Cancer Care Model, therefore, engages both personal and professional support networks to be more informed on LGBTQ2S+ oncology and become more representative for queer people, allowing for more consolidated and holistic care that cultivates positive coping mechanisms while negating the need to rely on maladaptive mechanisms.

The present study has the potential to inform survivorship treatment programs for LGBTQ2S+ survivors of cancer to optimize their recovery, which will be increasingly relevant as the LGBTQ2S+ survivor population grows nationwide. While this can be at a more systemic policy level, there is the hope that, with connections at Queering Cancer, Rainbow Health Ontario, and other LGBTQ2S+ cancer networks, findings of successful coping mechanisms and the care model developed can be shared with LGBTQ2S+ survivors of cancer directly, so that they may explore these options in their survivorship journey. Also, the literature on resilience and coping in LGBTQ2S+ survivors of cancer is very limited and this study provides novel research in this nascent field. It would be beneficial to have future research evaluating more specific sub-groups within the LGBTQ2S+ community or intervention research that applies this model or other LGBTQ2S+ support care models in practice.

### 4.4. Strengths and Limitations

The present study’s conclusions are novel, as they include non-sex-specific or non-gendered survivors of cancer. LGBTQ2S+ oncology studies have often focused on breast, uterine, and cervical cancer in women-identifying patients and on prostate cancer in male-identifying patients [34]. While these are the most sex-specific cancers and therein may be more relevant for studying LGBTQ2S+ people with cancer, there is still importance in assessing survivors who experience other types of cancer as they make up a majority of LGBTQ2S+ cancer survivors [5]. Another key strength of the present study is the use of a strengths-focused approach. To our knowledge, this is the first study to use such a framework in an LGBTQ2S+ cancer population. Having a strengths-focused approach has positive ramifications on LGBTQ2S+ communities and may allow for greater translation and uptake of any findings presented in the research [12]. Because the focus of this research is to provide insight and findings to inform LGBTQ2S+ cancer networks, having this framing of the research is important.

While the present study was reasonably ethnically diverse, it was limited to English-speaking LGBTQ2S+ survivors of cancer. This may limit the reach of this research to Francophones or minority groups in Canada who do not speak English. Moreover, geographically, concerns of a predominantly urban and Ontario-based sample dominated the results. LGBTQ2S+ populations experience higher rates of discrimination and lower access to health care services in rural settings [35], with this group therein an important demographic that are missed in this study and who should be explored in future research. Additionally, some of the participants in the study were less than 6 months into their survivorship journey. This may limit the breadth of survivorship that they have experienced and therein limit the experiences they can share for the study. Also, there was limited knowledge about the tumor site and disease stage, which could influence survivors’ experiences. Despite this, all participants in the present study identified strongly with their identity as a survivor of cancer, and all spoke to elements of their cancer journey starting as far back as their diagnosis, therein providing meaningful contributions to the study. Another limitation was the smaller sample size. While a sample of this size is adequate for qualitative IPA work, the sample still homogenizes the whole 2SLGBTQ2S+ community. There is a milieu of identities even within the LGBTQ2S+ community, who may all have unique needs within cancer care, and that cannot be accurately captured for each group in this work. For that, more specific groups will have to be studied in further detail. As well as this, this study’s model and findings, while informative, must be interpreted in the context of the sample size.

## 5. Conclusions

The present intersectional study explored the experiences of LGBTQ2S+ survivors surrounding resilience and coping, which addresses the first aim. To our second aim, it was seen that both positive coping mechanisms and negative mechanisms were part of how LGBTQ2S+ people with cancers coped during and after treatment; these coping mechanisms reflected those used by LGBTQ2S+ people with cancer when they were initially coming out, termed the ‘Second Coming-Out’ phenomenon. The mechanisms used, however, were adapted to a cancer context, such as oncology-specific therapy in place of standard therapy and queer representation in cancer media instead of queer representation in general media. These findings helped to inform the novel LGBTQ2S+ Cancer Care model, where the patient is centered around personal and professional networks that recognize LGBTQ2S+ issues, which fulfills the third aim. This work, therefore, uplifts the strengths of LGBTQ2S+ people with cancer to help foster resilience and improve their quality of life and sense of self.

“But just that having cancer is alienating from the world around you. Because when you’re in the midst of it, no one else can really understand it because it’s so very, very isolating. And then being queer is, can also involve a lot of that feeling of being isolated. And so, when you find people who care or understand or are interested, that’s just such a good feeling. And so, thank you for doing this research, because that’s how this feels.”

(S2)

## Figures and Tables

**Table 1:** Demographic and clinical characteristics.

	*n*	%
**Ethnicity** *		
White	4	40
First Nations	1	10
Black	3	30
South Asian	1	10
East Asian	1	10
South-East Asian	1	10
**First language**		
English	9	90
Chinese	1	10
**Place of birth**		
Canada	8	80
United States	1	10
Asia	1	10
**Highest level of education**		
High school	1	10
College/trade school	2	20
University (undergraduate)	5	50
University (professional/postgraduate/doctoral)	2	20
**Income**		
Less than $30,000	2	20
$30,001–50,000	2	20
$50,001–80,000	4	40
$80,001–120,000	2	20
**Main source of income**		
Employment/business	6	60
Retirement/pension	1	10
Family support	2	20
**Marital status**		
Single (never legally married)	4	40
Married	4	40
Common-law	1	10
Divorced	1	10
**Living situation**		
Living with a partner	6	60
Living with family	2	20
Living alone	2	20
**Community**		
Urban	8	80
Rural	2	20
**Province of residence**		
Alberta	2	20
New Brunswick	1	10
Ontario	7	70
**Private health insurance**		
Yes	7	70
No	3	30

* Participants could select multiple responses. All (*n* = 10) participants came out during adolescence. All participants experienced adverse experiences during the coming out process due to stigma surrounding LGBTQ2S+ issues, cultural barriers, and a general lack of knowledge about queer issues from family and friends.

## Data Availability

The data presented in this study are available upon request from the corresponding author. The data are not publicly available due to the small sample size.

## Appendix A

Demographics Questionnaire:

(1) What is your current age in years and date of birth?
(2) What is your gender?
  - Man
  - Woman
  - Non-binary
  - Transgender
  - Genderqueer
  - Genderfluid
  - Agender
  - Two-Spirit
  - Other (please specify)
  - Prefer not to answer
(3) What is your sexual orientation?
  - Heterosexual
  - Lesbian
  - Gay
  - Bisexual
  - Queer
  - Asexual
  - Pansexual
  - Demisexual
  - Questioning
  - Other (please specify)
  - Prefer not to answer
(4) What is your ethnicity?
  - Caucasian/White
  - First Nations (North American Indian)
  - African American/Black
  - South Asian/Chinese/Filipino
  - Indian subcontinent
  - Arab
  - Other (please specify)
(5) What is your citizenship?
  - Canadian citizen or permanent resident
  - Temporary resident in Canada
(6) What is your first language?
  - English
  - Other (specify)
(7) Where were you born?
  - Canada
  - United States
  - Africa (please specify)
  - Asia (please specify)
  - Europe (please specify)
  - Australia/New Zealand
(8) What is your highest level of education?
  - Elementary to high school
  - Trade school
  - Community College
  - University (undergraduate)
  - University (professional/postgraduate/doctoral)
(9) What is your occupation?
(10) What is your average annual income?
  - <30,000
  - 30,000–50,000
  - 51,000–80,000
  - 81,000–120,000
  - >120,000
(11) What is your main source of income?
  - Employment/business/investment
  - Pension/retirement
  - Social assistance
  - Family support (spouse, parents)
  - Others (please specify)
(12) What is your marital status?
  - Single (never legally married)
  - Married
  - Common-law
  - Divorced
  - Separated
  - Widowed
(13) Which of the following best describes your living arrangements?
  - Living with a partner (e.g., boy/girlfriend, spouse)
  - Living with roommate(s)
  - Living with family (e.g., sibling(s), parent(s))
  - Living alone
(14) What type of area do you live in?
  - Metropolitan
  - Regional/remote
(15) Do you have private health insurance?
  - Yes (employer-sponsored/self-sponsored)
  - No

## Appendix B

Semi-structured interview guide:

1. Generally speaking how have your experiences in medical settings been?
  - Do you feel your sexual or gender identity has influenced this? Why or why not?
2. Please describe your experiences during the cancer treatment and recovery process?
  - Do you feel your sexual or gender identity has influenced this? Why or why not? If why, how has it influenced the process?
3. How has cancer and cancer treatment influenced your own identity or perceptions of your identity? If no influence, why not?
  - How has cancer survivorship been part of your identity?
  - How have you understood or conceptualized having these multiple identities?
4. How would you define coping and resilience, particularly in a cancer context?
5. How do you feel coping or resilience was part of your cancer journey?
  - What coping mechanisms did you use in your cancer journey?
  - How do you feel your sexual or gender identity influenced your coping process? If not, why not?
6. What are supports for coping or resilience you would have liked to see during your cancer process?
  - How do you feel your cancer experience could have been improved?

## Appendix C

SS Reflexivity Statement
Reflexivity statements serve as an opportunity for researchers to acknowledge the position they arrive to the research with. Our position in the world, through our identity or background, can influence how we act, think, and research. I come to this research project as an LGBTQ2S+-identifying person, recognizing my own personal connection to the community and my own background as an advocate for LGBTQ2S+ health. I do not, however, have any background as a cancer survivor and in particular have not previously been acquainted with the LGBTQ2S+ oncology community. In this way, I come to the research with some knowledge on the subject but a complete lack of knowledge in other aspects. My own personal education in public and population health, however, has primed me to recognize the social determinants of health and how socially relevant identities (such as LGBTQ2S+ status) influence health. My work has consistently been guided by principles of health equity and I bring that lens of social health and health equity into all my research work. This project is no exception. I recognize that, particularly in qualitative work, there is the opportunity for my own personal beliefs to spill into the work. By first recognizing my position, as is being done in this reflexivity statement, I can attempt to recognize these understandings and fully analyze the findings through the perspectives of the participants themselves.
AS Reflexivity Statement
Acknowledging one’s positionality is an essential practice in qualitative research and I recognize that the construction of the present study, my conceptualization of the data, and the act of writing and refining this manuscript is, in part, shaped by my lived experience and personal identities. As a white cisgender bisexual woman, I carry my lived experience navigating Canadian culture and my own internal insecurities, uncertainties, and pride surrounding my identity into the research we have produced; in many ways, my proximity to this research motivated me to engage with the data in a truly immersive way; however, it also may have impacted what concepts within the data stood out to me. Moreover, my education in health science often came through the lens of critical disability theory, and consequently, when considering health provision, I typically focus on structural barriers and consider the ways in which society shapes individual experience. I also recognize that the LGBTQ2S+ community is highly heterogeneous and the experiences of myself, as a bisexual woman, can be extremely different from other members of the community. Moreover, LGBTQ2S+ identities are often inextricably intersectional, and thus, the privileged position I hold as a white person who was born in an urban community to a wealthy family separates my experience from that of many other LGBTQ2S+ people in the present study and within the broader community. I also have never been diagnosed with cancer, and thus have not faced the physical or psychosocial challenges of cancer, treatment, survivorship, or grappled with the choice of self-disclosure in these settings. I am grateful to have worked alongside my co-authors on this study, and to have had the opportunity for the iterative and reflexive nature of this project to change my relationship with my own identities.
